# Yttrium-90 Radioembolization as a Safe and Effective Treatment Option for Hepatic Uveal Melanoma Metastases

**DOI:** 10.1007/s00270-026-04373-y

**Published:** 2026-02-20

**Authors:** Jason R. Ni, Matthew Quirk, Bartosz Chmielowski, Siddharth A. Padia

**Affiliations:** 1 Division of Interventional Radiology, Department of Radiology, David Geffen School of Medicine, University of California Los Angeles, 757 Westwood Plaza, Suite 2125, Los Angeles, CA 90095 USA; 2 Division of Hematology-Medical Oncology, Jonsson Comprehensive Cancer Center, University of California Los Angeles, Los Angeles, CA USA

**Keywords:** Uveal melanoma, Hepatic metastases, Yttrium-90 radioembolization, Selective internal radiation therapy

## Abstract

**Purpose:**

Assessing the safety and efficacy of Yttrium-90 (^90^Y) segmentectomy and lobar radioembolization as a treatment option for patients with hepatic uveal melanoma metastases.

**Materials and Methods:**

An IRB-approved retrospective study of patients with liver metastases from uveal melanoma treated by radioembolization from 2017 to 2023 was performed. 25 patients (9 males, 16 females) with a median age at metastatic diagnosis of 62 years (range 30–78) underwent a total of 52 glass radioembolization procedures. Date of diagnosis and metastasis, tumor characteristics, concomitant therapies, treatment dosage, treatment related toxicities, survival, and imaging response were collected and reviewed. Imaging response was based on RECIST criteria as well as complete or partial radiographic response of the index tumor.

**Results:**

Mean index tumor size was 4.1 ± 1.0 cm (range 0.9–20). Complete radiographic response (n = 32) or partial response (n = 17) was achieved in 49/52 (94%) treatments. Segmental radioembolization showed higher rates of complete radiographic response compared to lobar radioembolization (78% vs. 35%, *p* < 0.05). Index tumor size was an independent factor in achieving optimal response rates for radiation segmentectomy (*p* < 0.05). Overall RECIST disease control rate was 84% (21/25). Median progression free survival and median overall survival from index treatment was 8.0 months (range 0.7–49.0) and 20.3 months (range 1.6–53.1), respectively. One year survival was 76% (19/25). Twelve mild Grade 1a clinical toxicities were reported (48%).

**Conclusion:**

This study supports ^90^Y radioembolization as a safe and effective treatment option for patients with hepatic metastatic uveal melanoma, with radiation segmentectomy achieving high rates of radiographic response and disease control rates.

**Graphical Abstract:**

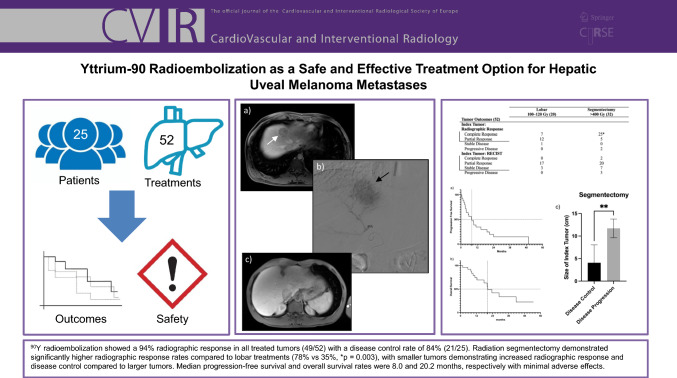

## Introduction

Uveal melanoma accounts for 3–5% of melanoma cases in the United States and nearly 50% of patients develop metastatic disease [1–3]*.* Unlike cutaneous melanoma which commonly spreads to the lung and brain, uveal melanoma is characterized by liver-predominant metastasis with hepatic involvement in over 90% of cases [4, 5]. Prognosis following hepatic metastases are poor, with a median survival of < 6 months and one-year survival of 10–15% [5–7]. Systemic immunotherapies, such as tebentafusp and nivolumab/ipilimumab, are currently offered to patients with metastatic disease [8], but recent studies show that response rates with these therapies are discouraging with a mean progression free survival of 3 months [9–11].

Current National Comprehensive Cancer Network (NCCN) guidelines recommend clinical trial enrollment for patients with metastatic uveal melanoma, reflecting the lack of standard-of-care therapy [12]. In the United States, these include tebentafusp for HLA-A*02:01–positive patients [13] and more recently, percutaneous hepatic perfusion (PHP) with melphalan [14]. Recent studies have shown promising results when combining immunotherapy with PHP [15, 16], but there continues to be a subset of patients with multifocal and oligometastatic disease that may not tolerate or qualify for these treatment options.

Despite the lack of an FDA indication, liver-directed locoregional therapies including: transarterial chemoembolization (TACE), percutaneous ablation, surgical resection, and ^90^Y radioembolization, are offered to control metastatic disease progression in these patients. TACE may achieve temporary disease stabilization, but durability is often limited [3, 17]. Similarly, percutaneous ablation and surgical resection offer high rates of local tumor control but are also associated with high rates of recurrence [3, 17]. ^90^Y radioembolization has shown promising efficacy in the setting of multifocal, bilobar disease in multiple malignancies [18–32], and there is growing interest in radiation segmentectomy for the treatment of patients with solitary or oligometastatic disease [33]. Collectively, these outcomes demonstrate the continued need for safety and efficacy data of locoregional treatments in establishing treatment strategies in patients with metastatic disease.

This purpose of this study is to evaluate the clinical outcomes and safety profile of ^90^Y segmentectomy and lobar radioembolization in patients with hepatic metastases from uveal melanoma.

## Materials and Methods

An institutional review board approved retrospective study of patients with uveal melanoma hepatic metastases treated by ^90^Y radioembolization from January 2017 to May 2023 was performed. 25 patients (9 males, 16 females) with a median age at metastatic diagnosis of 62 years (range, 30–78) were treated.

Inclusion criteria for radioembolization included: liver-only or liver-dominant metastatic disease (as defined as metastatic disease with minimal extrahepatic involvement), life expectancy > 3 months, ECOG performance status 0–2, and adequate liver function (i.e. laboratory values bilirubin < 2 mg/dL, albumin > 3 g/dL, AST/ALT < 5 × upper limit of normal, 200 U/L and 240 U/L, respectively). Patients with liver-dominant metastases were considered to have their extrahepatic disease well-controlled. Exclusion criteria included: tumor burden > 50% of the liver parenchyma, uncorrectable arterial supply to GI tract seen on angiography or Tc99m-MAA scan, lung shunt resulting in > 30 Gy dose to lungs per treatment or > 50 Gy cumulatively, biliary obstruction, or biliary-enteric anastomoses.

### Baseline Imaging

Pre-treatment tumor burden was evaluated with cross-sectional imaging and the number of tumors targeted by ^90^Y was recorded. Tumor burden was estimated by radiographic visualization and separated into percentage of liver involvement, either ≥ 20% or < 20%. Liver only versus liver-dominant disease and tumor morphology (i.e. nodular, well-circumscribed lesions, vs. infiltrative lesions, without clear borders) were recorded.

### ^99m^Tc Mapping Angiogram and ^90^Y Radioembolization Treatments

Initial mapping angiograms and ^90^Y radioembolization treatments were performed by interventional radiologists with IR/DR board certification. All patients underwent abdominal visceral mapping angiograms of the intrahepatic and relevant extrahepatic arterial anatomy before radioisotope administration. If necessary, prophylactic coil embolization of nontarget extrahepatic vessels was performed. ^99m^Tc –macroaggregated albumin was injected into the hepatic arteries and single-photon emission CT was performed to assess for unintentional delivery of radioactive microspheres to extrahepatic structures and to estimate the percentage of hepatic shunting to the lungs. Radioembolization was performed with ^90^Y glass microspheres (TheraSphere®, Boston Scientific, USA).

Focal tumors were treated with segmental ablative dosing, with target doses ≥ 400 Gy to the perfused volume. In the absence of historical data regarding uveal melanoma, this dosing is based on the LEGACY study, which showed that doses ≥ 400 Gy achieve high rates of complete tumor necrosis, albeit in HCC [34]. Patients with multifocal disease were treated in a lobar or bilobar fashion with a target dose of 100–120 Gy to the perfused volume. Patients with bilobar disease underwent sequential treatments to each lobe separated by 1–2 months in order to allow for functional and hepatic recovery from the initial treatment in according to the most recent radioembolization standards of practice guidelines recommended by the Society of Interventional Radiology [20]

### Patient Assessment and Treatment Response

Patients were evaluated for acute toxicity and metastatic progression one month after radioembolization therapy, and then every three months thereafter. Post-procedural adverse effects were classified according to the CIRSE Standards of Practice for the Classification of Complications up to 120 days after the date of procedure [35].

Contrast-enhanced CT and/or MRI scans of the chest, abdomen, and pelvis were obtained 2 months after treatment. Radiographic response of the index tumor after each treatment at the date of best imaging response, and disease progression were characterized by two board-certified radiologists. Radiographic response was defined as absence of contrast enhancement within the entire tumor (complete response) or part of the tumor (partial response) on imaging. Tumor progression was assessed by the investigators of the study using the Response Evaluation Criteria in Solid Tumors (RECIST), version 1.1 [36]. Disease control was defined as: complete response + partial response + stable disease.

Adverse events were classified using Common Terminology Criteria for Adverse Events (CTCAE), Version 5 up to 120 days after each treatment [37]. Clinical toxicities were defined as adverse events directly experienced and reported by the patient. Biochemical toxicities were defined as adverse events detected through laboratory abnormalities, not directly experienced or reported by the patient.

Survival rate was calculated one year from the date of initial radioembolization. The median overall survival period was measured from initial radioembolization to death, or date of last clinic follow up. Progression free survival of hepatic metastases was measured from initial radioembolization to confirmation of progression of hepatic metastases at diagnostic imaging, or death.

### Statistical Analysis

Overall survival and progression-free survival was estimated with Kaplan–Meier analysis. Log-rank test was used to conduct univariate analysis of survival differences between subgroups. Radiographic response of the index tumor and overall RECIST response were analyzed using Fischer’s exact test. Size of tumor and disease control rate of responders versus non-responders were compared using an unpaired *t*-test. A value of *p* < 0.05 was considered significant for all analyses. Statistical analyses were performed using GraphPad Prism version 10.5.0 (GraphPad Software, Boston, Massachusetts).

## Results

### Descriptive Results

Twenty-five patients were treated with 52 radioembolization procedures from January 2017 to May 2023 (Table 1). Eighteen patients (72%) had a hepatic tumor burden of < 20% and 7 patients (28%) had metastases involving ≥ 20% of the liver. 17 patients (68%) had liver-only disease and 8 patients (32%) had extrahepatic, but liver-dominant, disease. 20 patients (80%) had well-circumscribed tumors and 5 patients (20%) had infiltrative tumors. Eleven patients were HLA A*02:01-positive, 9 of which were previously treated with immunotherapy. 21 patients (84%) were on concomitant immunotherapy during radioembolization: the most common regimens were nivolumab ± ipilimumab (7 patients) or tebentafusp (6 patients). Patients who were on systemic immunotherapy continued treatment during the Y90 procedures. Three patients (12%) underwent percutaneous microwave ablation for focal tumors prior to radioembolization. No patients were previously treated with other transarterial directed therapies (i.e. TACE or PHP).

**Table 1:** Patient and treatment characteristics

	# (range)
*Demographics*	
Total patients	25
Male	9
Female	16
Median age @ primary diagnosis (years)	59 (29–76)
Median age @ metastatic diagnosis (years)	62 (30–78)
Median difference between primary and metastatic diagnosis (months)	33.3 (2.3–118)
*Pre-treatment patient characteristics*	
Median ECOG performance	0 (0–3)
Median total bilirubin (mg/dL)	0.4 (0.2–0.9)
Median albumin (g/dL)	4.2 (2.6–4.7)
HLA A*02:01 status	
Yes	11
No/unknown	14
Concomitant systemic therapy	
Yes	21
No	4
Extrahepatic disease	
Yes	8
No	17
*Pre-treatment tumor imaging factors (25 Patients)*	
% Liver involved	
< 20%	18
≥ 20%	7
Tumor morphology	
Nodular	20
Infiltrative	5
Mean index tumor size (cm)	4.8 (0.9–20)
*Post-procedure tumor factors (52 Treatments)*	
Treatment type	
Lobar	20
Segment	32
Mean # of procedures per patient	2 (1–4)
# of tumors treated per y90 session	
< 5	26
≥ 5	26

20 lobar (100–120 Gy) and 32 segmental treatments (> 400 Gy) were performed. The median time elapsed between hepatic involvement to treatment was 7.1 months (range, 0.9–52.2). The median age at radioembolization was 64 years (range, 31–81). Seven patients received one radioembolization treatment (either lobar or segmentectomy) and 18 patients received more than one radioembolization treatment. The median number of procedures per patient was 2 (range, 1–4). Thirteen tumors were treated in the caudate lobe (segment 1), 54 tumors were treated in the left hepatic lobe (segments 2–4) and 97 tumors were treated in the right hepatic lobe (segments 5–8). The mean index tumor size treated was 4.1 cm and median index tumor size treated was 2.75 cm (range 0.9–20 cm).

**Fig. 1:**
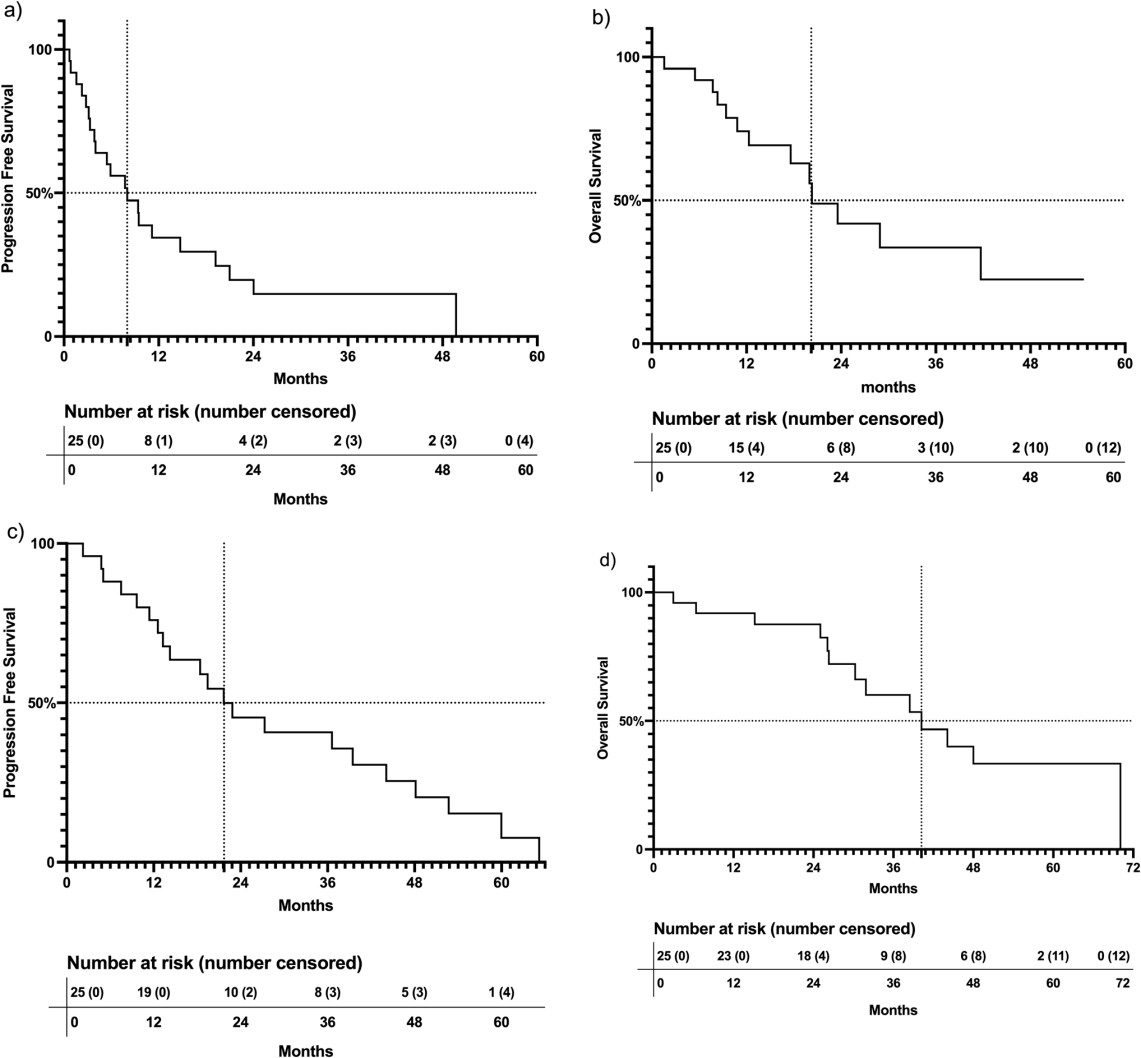
Kaplan Meier Survival Curves at date of first ^90^Y treatment and date of first metastatic disease. **a** Median progression free survival from the date of first ^90^Y treatment was 8.0 months (range 0.7–49.0). **b** Median overall survival from the date of first.^90^Y treatment was 20.2 months (range 1.6–54.7). **c** Median progression free survival from the date of first metastatic disease was 21.7 months (range 2.2–63.0). **d** Median overall survival from the date of first metastatic disease was 40.2 months (range 3.0–67.1)

Clinical follow up ranged from 0.8 to 53.9 months (median, 15.5 months). Median progression free survival was 8.0 months from the date of first treatment (Fig. 1a, range 0.7–49.0). Median overall survival was 20.2 months from the date of first treatment (Fig. 1b, range 1.6–54.7). The one-year survival rate of patients after their first treatment was 76% (19/25). There were no treatment-related deaths.

Thirteen patients died before the study end date from sequalae of hepatic tumor progression. All 5 patients with infiltrative disease died and demonstrated disease progression within one year (median 3.3 months, range 0.9–8.6 months). Individuals who died had a higher initial tumor burden (6/13 patients with ≥ 20% liver involvement, *p* = 0.07) and number of treated tumors with radioembolization (8/13 patients with ≥ 5 hepatic tumors, *p* = 0.37).

### Tumor Outcomes: Radiographic Response

**Fig. 2:**
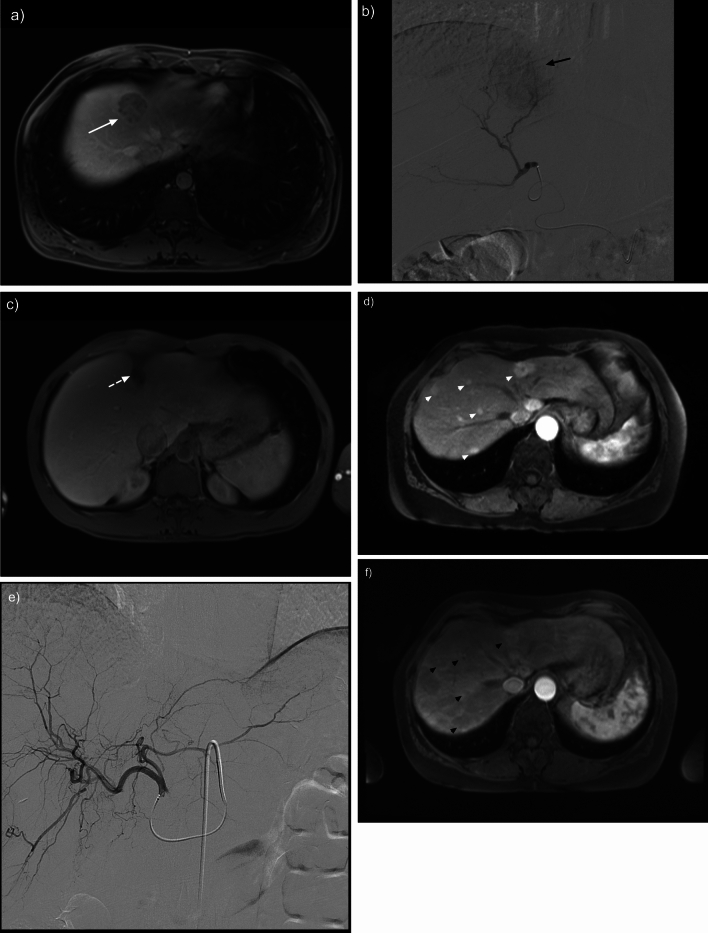
Representative images of index tumors status post ^90^Y radioembolization. Index tumor segmentectomy: **a** T1 weighted contrast enhanced MRI demonstrating an enhancing lesion in segment IV (white arrow). **b** Representative angiogram demonstrating ^90^Y segment radioembolization (black arrow). **c** T1 weighted contrast enhanced MRI demonstrating complete radiographic response of the treated lesion 5 years later (white dotted arrow). Index tumor lobar treatment: **d** T1 weighted contrast enhanced MRI demonstrating multiple hypervascular tumors in the right hepatic lobe (white arrowheads). **e** Representative angiogram image demonstrating right sided ^90^Y lobar radioembolization. Note, there is a replaced right hepatic artery arising from the superior mesenteric artery. **f** T1 weighted contrast enhanced MRI demonstrating partial radiographic response 3 months later (black arrowheads). This patient later underwent additional ^90^Y lobar radioembolization treatments of the left hepatic lobe

Complete radiographic response (n = 32) or partial response (n = 17) was achieved in 94% of treatments (49/52). Radiation segmentectomy resulted in complete or partial radiographic response of 94% of index tumors treated (30/32) and lobar treated patients demonstrated 95% response (19/20) (Table 2). Patients undergoing radiation segmentectomy demonstrated a higher rate of complete radiographic response compared to lobar patients, 78% versus 25% (Table 2, *p* = 0.0–3). Representative images of complete and partial radiographic response are provided in Fig. 2.

**Table 2:** Index tumor outcomes: Radiographic Response and Response Evaluation Criteria in Solid Tumors (RECIST) of the lobar and segment treated groups

Tumor outcomes (52)	Lobar 100–120 Gy (20)	Segmentectomy > 400 Gy (32)
Index tumor: radiographic response		
Complete response	7	25*
Partial response	12	5
Stable disease	1	0
Progressive disease	0	2
Index tumor: RECIST		
Complete response	0	2
Partial response	17	20
Stable disease	3	7
Progressive disease	0	3

Statistically significant difference of radiographic response was found between segmentectomy and lobar treated groups (*p = 0.003)

**Fig. 3:**
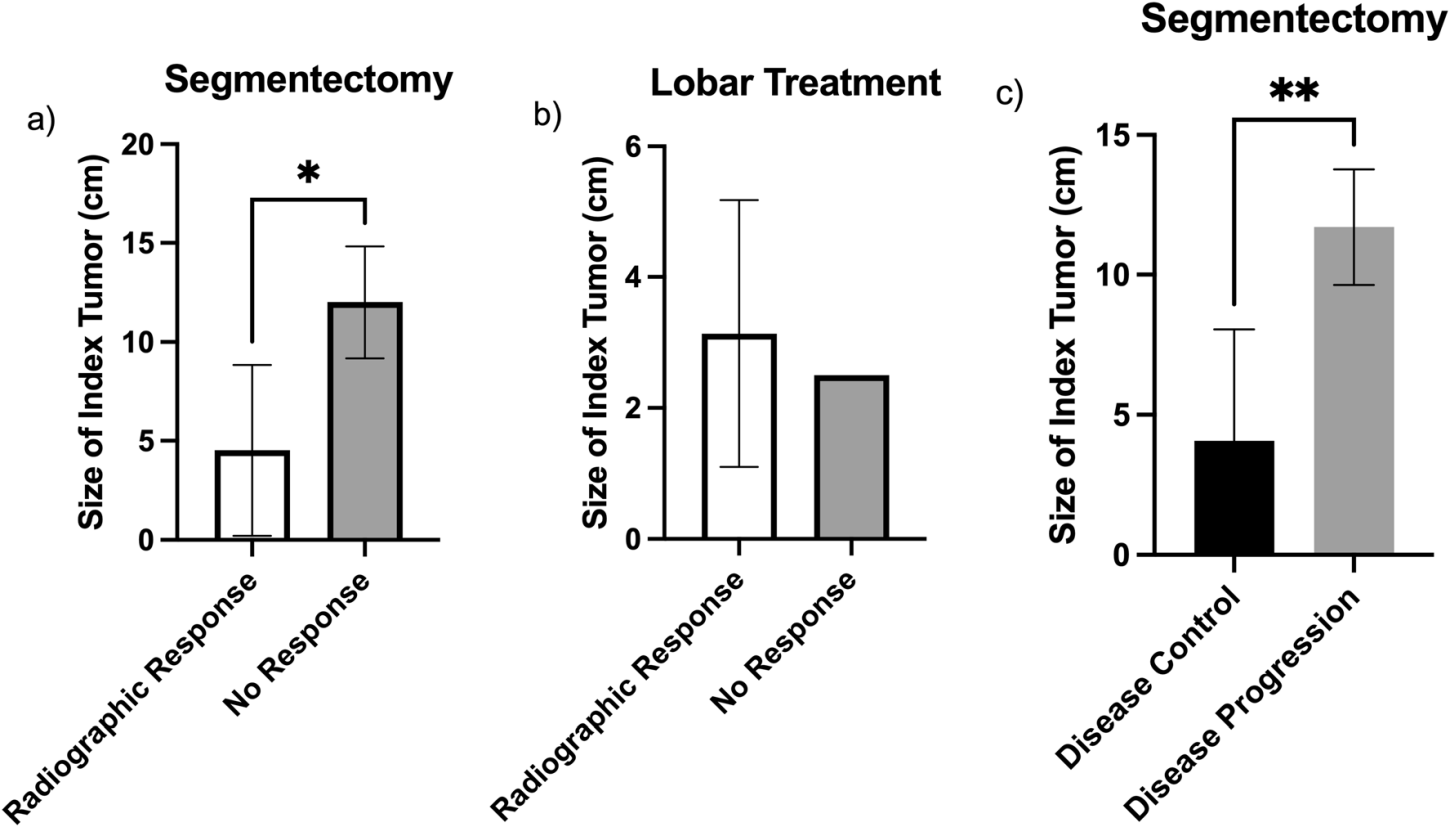
Effect of Pre-Treatment Tumor Size on Radiographic Response or RECIST Response. **a** Statistically significant difference in pre-treatment tumor size between tumors showing radiographic response after radiation segmentectomy versus those without, by unpaired *t*-test (*p* = 0.02). **b** No statistically significant difference in pre-treatment tumor size between tumors showing radiographic response after lobar treatment versus those without (*p* = 0.76). **c** Statistically significant difference in pre-treatment tumor size between tumors demonstrating disease control after radiation segmentectomy versus those without, by unpaired *t*-test (*p* = 0.003). RECIST response: lobar treatment was not graphed as all treatments demonstrated disease control

Smaller tumors demonstrated higher rates of radiographic response compared to larger tumors in the segmentectomy cohort. Tumors with a mean pre-treatment size of 4.5 ± 4.3 cm demonstrated increased rates of radiographic response compared to 12.0 ± 2.8 cm in the non-response group (Fig. 3a, *p* = 0.02). Similarly, mean pre-treatment size of lobar treated tumors demonstrating radiographic response was 3.1 ± 2.0 cm. One tumor measuring 2.5 cm exhibited no evidence of radiographic response at best imaging response (Fig. 3b).

### Tumor Outcomes: RECIST Response

91% of the segmentectomy group (29/32) and 100% of the lobar group (20/20) demonstrated disease control. Two complete RECIST responses were demonstrated in the radiation segmentectomy group while no lobar treatments demonstrated a complete response (Table 2). Progressive disease was seen after three treatments in the segmentectomy group.

Smaller tumors showed better rates of disease control compared to larger tumors when treated with segmentectomy. The mean pre-treatment size of tumors treated with segmentectomy demonstrating disease control was 4.1 ± 4.0 cm compared to 11.7 ± 2.1 cm of tumors demonstrating disease progression (Fig. 3c, *p* = 0.003). In the lobar treated group, disease control rates were achieved in all patients at the time of best imaging response regardless of tumor size.

### Adverse Effects

All radioembolization procedures were completed without complications. Grade 1a clinical toxicities were reported in 12 patients (48%) that required no further intervention, with abdominal pain being the most common (Table 3). There were no clinical Grade ≥ 3a toxicities. No patients met criteria for radioembolization induced liver disease after treatment. Two patients were clinically diagnosed and expectantly managed for post-embolization syndrome without complications. Biochemical toxicities were recorded across 7 patients (28%). This included 4 cases of transient transaminitis and one record of Grade 3a leukopenia requiring hospitalization and administration of prophylactic antibiotics (Table 3). This self-resolved with no complications. There were no differences in adverse effects between the lobar and segmentectomy groups.

**Table 3:** Clinical and Biochemical Adverse Event Profile (Common Terminology Criteria for Adverse Events, CTCAE Version 5.0). 12/25 (48%) of all patients experienced a mild clinical toxicity, the most common of which was abdominal pain (7/12, 58%)

All adverse events	Count (%)
Clinical toxicity	*n* = *12*
Abdominal pain	7 (58%)
Post embolization syndrome	2 (17%)
Fatigue	3 (25%)
Nausea/vomiting	3 (25%)
Fever	1 (8.3%)
*Any cause grade* ≥ *3a*	*0 (0%)*
Biochemical toxicity	*n* = *7*
Transaminitis	4 (57%)
Hypoalbuminemia	3 (43%)
Leukopenia	2 (29%)
Hyperbilirubinemia	1 (14%)
*Any cause grade* ≥ *3a*	*1 (14%)*
Portal hypertension (i.e. ascites, varices)	0 (0%)
Radioembolization-Induced Liver Disease (REILD)	0 (0%)

No CTCAE clinical toxicities Grade ≥ 3 were reported. 7/12 (58%) of all patients experienced a biochemical toxicity, the most common of which was transaminitis (4/7, 57%). One biochemical CTCAE Grade ≥ 3 toxicity was reported which resolved without complications

## Discussion

This study demonstrates that lobar and/or segmental radioembolization is effective in RECIST disease control with high rates of radiographic response seen on post-procedural imaging (94%, 49/52). Gonsalves et al. was the first to describe radioembolization as a treatment option for uveal melanoma hepatic metastases [18]. In their study, patients were treated with fractionated whole-liver or lobar radioembolization. They found that mean overall survival and progression free survival ranged from 19.2 months to 5.2 months, respectively with minimal toxicities and adverse events. The current study corroborates these findings with mean overall and progression free survival outcomes of 20.2 and 8.0 months, respectively.

Additionally, the current study demonstrates that radiation segmentectomy has higher rates of complete radiographic response of the index tumor compared to lobar treatments. This is likely due to the highly concentrated radiation dose delivered to the index tumor resulting in tissue necrosis. However, three instances of progressive disease were also found in this group. This may be a limitation of the study design as ^90^Y radioembolization was offered as a form of salvage therapy in the presence of increasing disease burden despite systemic therapy. Moreover, although the procedure may have been technically successful it may have not been technically effective. The tumors demonstrating progressive disease were all ≥ 10 cm at the time of segmentectomy and it is likely that the radiation threshold of necrosis was not achieved and higher rates of ablative dosing may be needed to achieve disease control in patients with more aggressive disease. Recent studies demonstrate that ablative doses > 1000 Gy may be safe in patients [38], but further research needs to be performed to establish a safe dose limit for patients with highly aggressive tumor biology, which is often seen in uveal melanoma [39, 40].Although radioembolization may lengthen overall and progression free survival, the majority of patients in the current study experienced disease progression over time (21/25), further substantiating the aggressive nature of uveal melanoma [1, 7, 18, 41, 42]. Similar to Gonsalves, et al., the current study also shows that patients with a higher tumor burden and more aggressive tumor characteristics (i.e. infiltrative, increased size) before radioembolization have a decreased treatment response compared to those without. This suggests that patients with less aggressive metastatic tumor characteristics (i.e. smaller tumor size, well-circumscribed, decreased tumor burden) may benefit more from radioembolization.

It should be noted that limitations of this study include a small sample size, heterogenous population, and data from a single institution. All patients underwent radioembolization without a comparison group and ^90^Y radioembolization was utilized as a form of salvage therapy for patients with more aggressive disease progression. In this regard, some patients received both lobar and segmental treatments during their disease course and the ability to analyze the synergistic effects between locoregional and systemic immunotherapy was limited. Because of this, individuals on different regimens of systemic therapy and prior liver directed treatments were included which may influence the outcomes. The type of systemic therapy between individuals varied throughout this study, but there was no difference in treatment response or adverse effects between patients on concomitant (21/25) versus non-concomitant therapies (4/25), or those with HLA A*02:01-positive disease. Moreover, there was an increased median overall survival from the initial diagnosis of metastatic disease of 40.2 months when compared to published data of the median overall survival of patients treated first line with tebentafusp of 22 months, pembrolizumab of 17 months, and the combination of nivolumab and ipilimumab of 12–16 months [10, 11, 43]. This demonstrates promising results of the synergistic nature of systemic therapy combined with locoregional radioembolization.

Recent data of the FDA approval of PHP with melphalan (Hepzato™, Delcath Systems, Inc, USA) describes a median overall survival of 20.5 months [44] in these patients, and recent findings from the CHOPIN trial combining PHP with systemic immunotherapy demonstrate promising results with a disease control rate of 85.7% (6/7 patients), similar to our control rate of 84%, with a median duration of response of 27.1 months [15]. However, it should be noted that the sample size in this trial was small and 4 out of 7 patients also experienced progressive disease, the earliest of which occurred at 8.9 months [15]. Moreover, Kolb, et al. retrospectively compared ^90^Y radioembolization with PHP which showed that although OS was significantly longer in patients undergoing PHP, median PFS was not significant between the two groups [45]. It is important to note that selection bias may play a role in these results as patient comorbidities (i.e. cardiovascular disease and functional status) may limit qualification for PHP. In this regard, the role of ^90^Y radioembolization remains important as not all patients may tolerate or qualify for PHP.

In conclusion, the current study demonstrates that ^90^Y radioembolization is a safe and effective treatment for patients with hepatic metastases from uveal melanoma. Both segmentectomy and lobar treatments lead to improved one year survival rates with low complication rates, with radiation segmentectomy demonstrating increased rates of complete radiographic tumor response.
